# Interactions between and Shared Molecular Mechanisms of Diabetic Peripheral Neuropathy and Obstructive Sleep Apnea in Type 2 Diabetes Patients

**DOI:** 10.1155/2018/3458615

**Published:** 2018-07-19

**Authors:** Hong Shen, Junrong Zhao, Ying Liu, Guangdong Sun

**Affiliations:** ^1^Department of Endocrinology, The Second Hospital of Jilin University, Changchun 130041, China; ^2^Department of Nephrology, The Second Hospital of Jilin University, Changchun 130041, China

## Abstract

Type 2 diabetes (T2D) accounts for about 90% of all diabetes patients and incurs a heavy global public health burden. Up to 50% of T2D patients will eventually develop neuropathy as T2D progresses. Diabetic peripheral neuropathy (DPN) is a common diabetic complication and one of the main causes of increased morbidity and mortality of T2D patients. Obstructive sleep apnea (OSA) affects over 15% of the general population and is associated with a higher prevalence of T2D. Growing evidence also indicates that OSA is highly prevalent in T2D patients probably due to diabetic peripheral neuropathy. However, the interrelations among diabetic peripheral neuropathy, OSA, and T2D hitherto have not been clearly elucidated. Numerous molecular mechanisms have been documented that underlie diabetic peripheral neuropathy and OSA, including oxidative stress, inflammation, endothelin-1, vascular endothelial growth factor (VEGF), accumulation of advanced glycation end products, protein kinase C (PKC) signaling, poly ADP ribose polymerase (PARP), nitrosative stress, plasminogen activator inhibitor-1, and vitamin D deficiency. In this review, we seek to illuminate the relationships among T2D, diabetic peripheral neuropathy, and OSA and how they interact with one another. In addition, we summarize and explain the shared molecular mechanisms involved in diabetic peripheral neuropathy and OSA for further mechanistic investigations and novel therapeutic strategies for attenuating and preventing the development and progression of diabetic peripheral neuropathy and OSA in T2D.

## 1. Introduction

Diabetes mellitus is a global disease with major public health implications and is predicted to affect 642 million persons by 2040 [1, 2]. Type 2 diabetes (T2D) accounts for 90–95% of all patients with diabetes and involves multiple systems and organs. Long-standing poorly controlled T2D ultimately leads to the development of microvascular complications, including neuropathy, nephropathy, and retinopathy, and macrovascular disease such as cerebrovascular and coronary artery diseases [3].

DPN is defined as “symmetrical, length-dependent sensorimotor polyneuropathy attributing to metabolic and microvessel alterations as a result of chronic hyperglycemia exposure and cardiovascular risk covariates” [4]. Fifty percent of T2D patients would eventually develop DPN and 20% of T2D patients have DPN at presentation [5]. However, DPN is mostly neglected as a diabetic complication. Furthermore, the pathophysiologic mechanism of DPN appears to be complex, involving the metabolic and ischemic pathways [3].

Recent evidence has suggested a link between obstructive sleep apnea (OSA) and DPN. OSA is one treatable type of sleep-disordered breathing characterized by episodes of complete or partial obstruction of the upper airway during sleep, resulting in recurrent episodes of apnea or hypopnea [6]. Growing evidence has shown that OSA is very common in T2D patients and probably associated with DPN [7, 8]. Several longitudinal studies and meta-analyses have indicated that OSA is a risk factor for T2D associated with insulin resistance and *β*-cell dysfunction [6] and could be a cause of ineffective treatment of T2D [9]. Conversely, T2D may also be a risk factor of OSA or worsen preexisting OSA. There is compelling evidence of an association between OSA and metabolic dysfunction, in particular, changes in glucose metabolism resulting in metabolic syndrome, glucose intolerance, and insulin resistance [10, 11]. Moreover, this association is independent of obesity, which is a common occurrence in patients with T2D and/or OSA [12].

As OSA and T2D frequently coexist, knowledge about the association between DPN and OSA in T2D could shed light on the pathogenesis of OSA and T2D in these patients. Recent data has suggested significant underappreciation of OSA in T2D patients, and the mechanisms underlying the link between OSA and DPN remain largely unelucidated. Dissecting the relationship between DPN and OSA is also clinically relevant as illumination of the connection between the two conditions may impact on both patient care and quality of life.

The primary aim of this review is to explore connections among OSA, DPN, and T2D and assess whether OSA and DPN could interact with each other in T2D patients. We also summarize the potential common molecular mechanisms whereby OSA and DPN could be linked in T2D, including the role of neuromodulators in DPN and OSA.

## 2. Complex Connections among OSA, DPN, and T2D

OSA is diagnosed when the apnea-hypopnea index (AHI) is ≥5/hr, together with such symptoms or signs as nocturnal gasping or choking events, witnessed habitual snoring, excessive daytime sleepiness, hypertension, nonrefreshing sleep, and congestive heart failure or AHI ≥ 15/hr without symptoms [13]. OSA affects about 14% of men and 5% of women, is highly prevalent in T2D patients [14, 15], and has also been linked to the development of incident T2D [12]. The relationship between OSA and T2D may be bidirectional given that DPN could influence central control of respiration and upper airway nerve reflex promoting sleep-disordered breathing. Several previous studies suggested an association between OSA and diabetic autonomic neuropathy [16, 17]. The development of autonomic neuropathy in T2D patients may affect upper airway innervation and collapsibility, ventilator drive, and central respiratory center reaction to hypercapnia stimulus, which contribute to the pathogenesis of OSA [6].

### 2.1. Bidirectional Link between OSA and T2D

Although substantial literature has established a link between OSA and T2D, there is lack of keen awareness of such an association, and clinically, T2D patients are not vigorously screened for OSA [6]. Furthermore, intermittent hypoxia and sleep fragmentation in OSA patients could independently induce intermediate disorders including sympathetic nervous system activation, systemic inflammation, oxidative stress, appetite-regulating hormone alterations, and hypothalamic-pituitary-adrenal axis activation, which in turn promote the development of insulin resistance, glucose intolerance, and ultimately T2D [18, 19]. There is also convincing evidence for an association between OSA and fasting insulin, glucose, and HbA1c levels independent of obesity although the exact pathophysiological mechanism underlying such a link still remains elusive [3]. Conversely, T2D could increase predisposition to, or accelerate progression of, OSA, possibly partially through the development of peripheral neuropathy [20]. It is not surprising, therefore, that there exists a link between OSA and T2D [21], in particular, considering the confounding effects of obesity and aging.

#### 2.1.1. OSA Affecting T2D

Longitudinal cohort studies, including 6 prospective cohort studies from different regions all over the world with a follow-up duration of 2.7–16 years, have shown a significant association between OSA and T2D [12, 22–31]. Some studies also showed that severity of OSA correlated with the presence of T2D. The prevalence of T2D in OSA patients was estimated to be 15–30% and may be even higher in severe OSA patients [24, 32–34]. However, after adjustment for body mass index (BMI) and other confounders, no correlation was found between OSA and T2D in some studies [24, 33, 34] while several other studies showed that increased OSA severity was robustly associated with increased HbA1c levels in T2D patients after adjustment for confounders [34–38]. This suggested that uncontrolled OSA may exacerbate the progression of T2D.

#### 2.1.2. T2D Affecting OSA

In spite of notable methodological limitations, several independent studies revealed a significantly higher prevalence (23–86%) of OSA in T2D patients versus the general population [27, 39], suggesting that T2D could be a risk factor for OSA. Scantly available data showed that T2D could worsen the progression of preexisting OSA [19], especially in patients with autonomic neuropathy [9]. In both clinic-based and community-based cohorts including T2D patients with diverse backgrounds, the prevalence of OSA was alarmingly elevated [7, 35, 36, 40–49] and insulin resistance could predict OSA development [39]. T2D affecting OSA is postulated to involve the disorders of the autonomic nervous system leading to sleep-disordered breathing. However, OSA is usually underdiagnosed in the majority of T2D patients in the primary care setting [50].

#### 2.1.3. DPN in T2D

DPN is more common in T2D patients, accounting for 60%–70% of individuals with diabetes [3] and contributes significantly to morbidity and mortality of diabetes patients [51]. DPN can be categorized into distal symmetric peripheral neuropathy and asymmetric (focal and multifocal) neuropathies (including multiple mononeuropathies and thoracic, lumbosacral, and cervical radiculoplexus neuropathies) [52]. A recent study evaluated the risk of neuropathy in 1414 T2D patients and found that diabetic women with altered sleep patterns had a higher risk of developing neuropathy [53]. The causes of DPN are multifactorial, including metabolic factors such as high fat, high glucose, and low insulin; autoimmune factors producing neurotoxic inflammation; neurovascular factors resulting in damage to vessels carrying nutrients and oxygen to the nerves; carpal tunnel syndrome at the wrists; and ulnar nerve entrapment at the elbows and lifestyle factors such as alcohol use and smoking [54]. However, the pathological progression of DPN is still unclear and it is essential to explore the potential molecular mechanism involved in DPN.

#### 2.1.4. Interaction between OSA and DPN in T2D Patients

OSA in diabetic patients is typically explained by obesity associated with T2D. Recently, OSA has been shown to be associated with DPN in T2D patients. The knowledge of such an association in T2D patients is of clinical implications. OSA and DPN in T2D could aggravate each other, resulting in a vicious circle, with even additive or synergistic health risks in T2D patients. A previous study examining the relationship between OSA and DPN found a fourfold increase in the odds of peripheral neuropathy in T2D patients with OSA compared with those without [7].

#### 2.1.5. DPN Affecting OSA

Diabetic neuropathies are a heterogeneous group of disorders affecting different parts of the nervous systems, including symmetrical polyneuropathies, autonomic neuropathy, and multifocal and focal neuropathies [19, 27]. Earlier data mainly focused on the effect of diabetic autonomic neuropathy on sleep-disordered breathing [55]. Diabetic autonomic neuropathy, a form of DPN [2, 56], could lead to ventilator dysfunction through impaired central control of breathing, leading to sleep-disordered breathing [57–60]. Diabetic neuropathy could increase upper airway collapsibility due to the destruction of the dilatory muscles of the larynx, which could aggravate OSA [19]. This mechanism is also observed in a peripheral neuropathy named Charcot-Marie-Tooth [61]. Another possible mechanism is sleep disturbance by painful peripheral neuropathy. A meta-analysis confirmed the relationship between OSA and diabetic neuropathy and revealed that OSA was documented more frequently in T2D patients with neuropathy [62]. Laboratory investigations have also shown that T2D patients with diabetic autonomic neuropathy are more likely to have OSA than those without [63], suggesting diabetic autonomic neuropathy as another explanation for the presence of OSA because it is diabetes specific. However, there are possible mechanisms as to why both diabetic autonomic neuropathy and DPN could lead to the progression of OSA. Patients with T2D and OSA are at risk of DPN [7]. Diabetes-related nocturia or pain from DPN could worsen sleep or cause sleep loss [54].

#### 2.1.6. OSA Affecting DPN

A recent study has implicated OSA as a risk of peripheral neuropathy; autonomic dysfunction risk could be positively correlated with severity of OSA [64]. Sleep-disordered breathing such as OSA has been documented in approximately 50–70% T2D patients and may contribute to diabetic neuropathy [41, 65]. Shorter or longer duration of sleep could increase the rate of complications such as DPN [66–68]. Evidence is scant supporting an association of OSA with DPN [8]. OSA is associated with nitrosative and oxidative stress as well as impaired microvascular regulation in T2D patients [7] and could lead to increase of insulin resistance and T2D, which in turn could elevate inflammatory markers and contribute to vascular complications [3]. Therefore, OSA-complicating T2D could facilitate the development and progression of microvascular complications including DPN. OSA has been shown to be independently associated with clinically evident DPN [7]. Robust data is now available supporting OSA as an independent risk for DPN development [3].

#### 2.1.7. Common Potential Molecular Mechanisms Involved in OSA and DPN

One study found that approximately 60% of patients with OSA and diabetes have peripheral neuropathy [69]. It has been postulated that advanced glycation end products (AGEs) and protein kinase C (PKC) could lead to microvascular complications including DPN [3, 7]. OSA and DPN in T2D patients may share molecular mechanisms underlying the development of both conditions as detailed below (also refer to Table 1 for further references).

#### 2.1.8. Oxidative Stress

Oxidative stress is characterized by excessive production of reactive oxygen species (ROS) overwhelming the body's antioxidative defenses [20]. Superoxide ion (O_2_^−^), nitric oxide (NO), and hydrogen peroxide (H_2_O_2_) are three radical ROS believed to mediate cellular degeneration in disease states [70]. ROS excess could inhibit insulin-induced energy substrate uptake in adipose and muscle tissues and damage pancreatic *β* cells [20, 71]. ROS could also suppress insulin secretion and worsen insulin sensitivity [72, 73]. Actually, cellular studies showed that intermittent hypoxia in OSA negatively affected *β*-cell death and proliferation, which could be attributed to excessive cellular oxidative stress [74].

Excessive oxidative stress is a well-recognized mechanism in the pathogenesis of DPN [75]. Previous studies implicated free lipid peroxidation product accumulation, increase in GSSG/GSH ratio, GSH depletion, and downregulation of superoxide dismutase (SOD) activity in DPN [76–79]. Further studies are needed to unravel the mechanisms of oxidative stress in the development and progression of DPN. In DPN, AGEs and PKC signaling directly alter cellular redox capacity through ROS formation [80].

Recent evidence suggests an association of OSA with high concentrations of ROS [71]. Upregulated oxidative stress has been repeatedly demonstrated in OSA patients [81–84] and could contribute to cerebrovascular, cardiovascular, and other morbidities of OSA. Increased prooxidant/antioxidant ratio could lead to oxidative stress associated with OSA, which is primarily attributed to decreased oxygen availability during apneic events and ROS formation during reoxygenation when breathing resumes [81]. Oxidative stress initiates a vicious circle in which it promotes inflammation and sympathetic activation, which in turn potentiates oxidative stress [85]. Accumulating evidence shows that increase in oxidative stress in OSA patients could contribute to hyperlipidemia, insulin resistance, T2D, and subsequent DPN [86].

Increased ROS production associated with hypoxia could be attributed to dysfunctional mitochondria, NADPH oxidase and xanthine oxidase, and uncoupling of nitric oxide synthase activation, leading to generation of ROS rather than nitric oxide (NO) [81, 87], ultimately injuring vital biomolecules and altering physiological signaling pathways [85]. Increased ROS levels due to intermittent hypoxia in mouse mitochondria could contribute to T2D development [88]. Recent studies have indicated that genetic polymorphisms of NADPH oxidase could affect oxidative stress levels in OSA patients [85]. The circulating levels of lipid peroxidation [89–92], DNA [91, 93], and oxidation products were increased in OSA patients and correlated with AHI severity [94, 95], which partially recovered by continuous positive airway pressure (CPAP) therapy.

#### 2.1.9. Inflammatory Markers

Diabetic patients have high circulating levels of inflammatory markers such as tumor necrosis factor-*α* (TNF-*α*), C-reactive protein (CRP), interleukin-6 (IL-6), and IL-8 [96, 97]. M1 macrophages in adipose tissues produce IL-6 and TNF-*α* [98, 99], which could lead to free fatty acid (FFA) release, ultimately resulting in impaired insulin signaling consequent of insulin resistance and metabolic dysfunction [11, 100].

T2D patients with DPN have markedly higher TNF-*α* levels than those without DPN and healthy persons, and high TNF-*α* may be an independent risk of DPN [101]. Furthermore, IL-6 levels are chronically elevated in T2D patients with DPN [102]. CRP is a sensitive biological marker of subclinical systemic inflammation related to insulin resistance, hyperglycemia, and overt T2D. T2D patients with DPN have noticeably higher CRP and TNF-*α* levels than those without DPN and normal subjects [103], suggesting a prominent role of inflammation in the development and progression of DPN. NF-*κ*B activation, which is involved in the pathogenesis of diabetic complications, especially DPN [104], has been identified in the endoneurium, epineurial vessels and perineurium in sural nerve biopsies of overt diabetic subjects [105].

Cytotoxic T lymphocytes could acquire an inflammatory phenotype in OSA patients [85]. CD8^+^ T cells exhibit elevated TNF-*α* levels and display enhanced cytotoxicities in an AHI severity-dependent manner [106, 107]. Cytotoxic *γδ* T cells express higher levels of proinflammatory cytokines, including TNF-*α* and IL-8, and lower anti-inflammatory IL-10 levels, suggesting that they could be implicated in atherogenesis in OSA patients [108]. In addition, several studies have found elevated levels of TNF-*α*, IL-6, and CRP in OSA patients [109–113]. Consistently, a proinflammatory state has been found in OSA patients [114]. Previous studies have shown that CPAP decreased the levels of IL-6, TNF-*α*, and CRP, leading to a reduction in vascular complications and inflammation [3, 111, 112]. Inflammatory markers that are implicated in DPN and OSA including TNF-*α*, IL-6, IL-8, and CRP are listed in Table 1.

Blood cells from OSA patients usually show a proinflammatory phenotype, which could lead to endothelial dysfunction, endothelial injury, atherosclerosis, and thrombosis [85]. Short-lived circulating neutrophils from moderate to severe OSA patients had a prolonged lifespan, which was associated with increased NF-*κ*B levels, decreased ratios of proapoptotic/antiapoptotic proteins, and a higher level of adhesion molecules [85]. Similar results were revealed in neutrophils from healthy controls exposed to intermittent hypoxia *in vitro* [115–118].

#### 2.1.10. Endothelin-1 (ET-1)

ET-1 is a potent vasoconstrictor and could also stimulate cellular proliferation. ET-1 contributes to endothelial abnormalities and imbalance of vasodilation and vasoconstriction in favor of the latter in diabetes. In diabetes patients, endoneurial microangiopathy, particularly basement membrane thickening, is related to clinical neuropathy. Several studies have implicated ET-1 as a novel risk factor for DPN, and ET-1 levels increased in DPN patients [119], while improvement of blood glucose did not affect ET-1 concentrations [120].

OSA patients exhibited augmented vasoconstrictive capacity due to ET-1 activation. Recurrent episodes of OSA increased ET-1 levels and blood pressure. Vasoconstrictor and mitogenic effects of ET-1 may be implicated in increased cardiovascular risk in OSA patients [120–122]. Other studies failed to show any ET-1 elevations in OSA patients compared with controls [123, 124]. In OSA rats mimicking intermittent hypoxia, ET-1 constrictor sensitivity rose in a PKC *δ*-dependent manner in the mesenteric arteries [125]. ET-1 has also been shown to be involved in ocular complications of OSA [126].

#### 2.1.11. Vascular Endothelial Growth Factor (VEGF)

VEGF stimulates angiogenesis by promoting vascular endothelial cell proliferation, migration, and proteolysis. Little is known regarding VEGF expression in human DPN. In diabetic rats, immunostaining of the sciatic nerve and dorsal root ganglion revealed high VEGF levels in cell bodies and nerve fibers [127]. The role of VEGF generated substantial interest in the therapy of neuropathy. VEGF administration has been shown to restore nerve blood flow, nerve conduction velocity, and nerve vessel number to normal in DPN [128].

VEGF is a hypoxia-sensitive glycoprotein. Plasma VEGF levels became elevated in severely hypoxic OSA patients and were correlated to the degree of nocturnal oxygen desaturation [129]. Similar results were found in both young and adult OSA patients, and plasma VEGF concentration was moderately correlated to OSA severity [130, 131]. Some other studies showed contrary results and found no correlation between plasma VEGF levels and severity of hypoxia [131–133].

#### 2.1.12. AGEs

AGEs are a complex group of compounds formed through nonenzymatic covalent bonding between reducing sugar and amine residues on lipids, proteins, or nucleic acids. They could also originate from exogenous sources including diet and tobacco smoke [134]. AGEs accumulate in local tissues because AGE-modified proteins are resistant to enzymatic degradation [135]. The role for glycation/glycoxidation in diabetic neuropathy has been extensively reviewed [136–139]. Several clinical studies implicated glycation in the pathogenesis of DPN secondary to T2D [140, 141]. Glycated myelin can stimulate macrophages to secrete proteases, which could contribute towards nerve demyelization in DPN [134, 138, 142]. Elevated AGE levels have been documented in the peripheral nerves of diabetic patients [138]. The AGE pathway is a main pathophysiologic mechanism in the development of DPN, and measures to reduce AGE formation could be useful in preserving nerve function in T2D patients [134].

AGEs are also increased in OSA, a condition in which increased systemic inflammation and oxidative stress are operationally activated [143, 144]. Intermittent hypoxia in OSA may induce AGE formation [145]. Plasma AGE levels were elevated and associated with insulin resistance in nondiabetic patients with OSA [146]. AGE accumulation in OSA may lead to diminution in early endothelial progenitor cells and endothelial repair capacity over time contributing to vascular pathogenesis [147].

#### 2.1.13. Protein Kinase C (PKC)

PKC is a family of enzymes involved in controlling the function of other proteins through phosphorylation of the OH groups on threonine and serine residues and has several isoforms. The role of PKC in the pathogenesis of DPN has been reviewed in detail [148]. There has been conflicting reports on PKC isozyme activity in dorsal root ganglion neurons in diabetic animals [148]. One report showed decreased PKC *α* mRNA levels in dorsal root ganglion neurons of diabetic rats compared with controls [149]. Another report showed higher aldose reductase expression in neurons, which reduced PKC *α* activity due to translocation from the membrane to cytosol, revealing a role of PKC *α* isoform in the hyperglycemic milieu [150]. Vascular tissues in diabetes showed increased PKC activity leading to increased permeability and dysfunction [151].

Proinflammatory cytokines such as TNF-*α* and IL-1*β* produced in the pulmonary arterial tissue were upregulated under hypoxic conditions in OSA patients, and the upregulation of these cytokines was dependent on PKC activation [152]. Intermittent hypoxia mimicking OSA could augment vasoconstriction mediated by PKC *δ* in a calcium-independent manner [125].

#### 2.1.14. Poly ADP Ribose Polymerase (PARP)

PARP becomes activated by oxidative stress-induced DNA damage, which plays an important role in the pathogenesis of DPN in T2D patients [153, 154]. A recent study has shown that PARP activation was independently associated with higher AHI secondary to oxidative stress in patients with OSA and T2D [8]. In addition, PARP activation provides another explanation for the longitudinal and cross-sectional associations between OSA and DPN in T2D patients [7, 155–157]. Intermittent hypoxia has been suggested to induce oxidative stress and PARP activation in nondiabetic rodents *in vitro* [158]. PARP inhibition could reverse DPN and neuropathy in diabetic rodents through alleviating oxidative stress [159–161].

#### 2.1.15. Nitrosative Stress

Nitrosative stress, which is marked by enhanced peroxynitrite formation, has been well documented in both clinical and experimental diabetic neuropathies [162]. A previous study has demonstrated higher serum nitrotyrosine levels in T2D patients with DPN, which is consistent with reports on experimental DPN [7], implicating nitrosative stress in DPN pathogenesis by reducing nerve perfusion and destroying vascular reactivity of epineurial arterioles [162, 163]. Nitrosative stress could also affect all the cell types in the peripheral nervous system, such as Schwann cells, and endothelial cells of the peripheral nerve, astrocytes, neurons, and oligodendrocytes of the spinal cord [159]. Nitrosative stress is related to the development of thermal hyper- and hypalgesia, tactile allodynia, mechanical hypalgesia, and small sensory nerve fiber degeneration [163]. Inhibition of nitrosative stress with PARP inhibitor or baicalein improved experimental neuropathy in diabetic rodent models [159, 164].

So far, a study has shown an association between OSA and nitrosative stress in T2D patients [7]. The obvious correlation between nocturnal/sleep-related hypoxemia and serum nitrotyrosine levels suggested that nitrosative stress was a potential mechanistic link between DPN and OSA. A previous study has shown greater endothelial expression of nitrotyrosine in OSA patients without T2D than OSA-free subjects regardless of central adiposity [165].

#### 2.1.16. Plasminogen Activator Inhibitor-1 (PAI-1)

PAI-1, a member of the serine protease inhibitor family, controls the fibrinolytic system by inhibiting urokinase and tissue-type plasminogen activators [103]. An earlier study indicated that diabetic neuropathy did not show any significant relationship with plasma PAI-1 levels in T2D patients [166]. A recent study has shown that PAI-1 levels were higher in DPN patients than normal subjects and T2D patients without DPN and PAI-1 was associated with DPN development from the perspective of inflammation, suggesting that PAI-1 and inflammatory markers such as TNF-*α* and CRP participated in the development and progression of DPN [103].

Increased PAI-1 activity was associated with sleep-disordered breathing, possibly contributing to increased vascular risk [167]. A previous study indicated that PAI-1 levels were significantly higher in OSA subjects than controls and PAI-1 positively correlated with AHI index [168]. CPAP therapy could significantly reduce PAI-1 levels. In OSA children, PAI-1 levels were significantly higher along with other inflammatory markers such as IL-6 and monocyte chemoattractant protein-1 (MCP-1) [169]. A recent study has shown that OSA patients had a higher median plasma PAI-1 level than controls and PAI-1 levels increased with OSA severity, suggesting that OSA could enhance prothrombotic activity [170]. OSA significantly correlated with PAI-1 concentration due to prothrombotic effects.

#### 2.1.17. Vitamin D Deficiency

Vitamin D, a steroid hormone with multifarious and extensive effect, could play a potential therapeutic role in attenuating the severity and progression of T2D [171, 172]. Growing evidence shows that low vitamin D level could contribute to pathogenesis of diabetes and its underlying diseases [173, 174]. Vitamin D deficiency has been implicated in the pathophysiology of DPN by impacting on nerve function [175].

Vitamin D deficiency may correlate with DPN in T2D patients [173, 176–180], with lower serum levels of 25(OH)D being independently associated with increased DPN in T2D patients [177, 181]. Vitamin D deficiency was also associated with development of neuropathy in T2D patients [182], as revealed in Caucasians and Asians with T2D by a recent meta-analysis [183]. Nerve growth factor (NGF), which is essential for primary nociceptive neuron development, was found by immunostaining to correlate with skin axon reflex vasodilation mediated by small sensory fibers in diabetic neuropathy patients [184]. Vitamin D is likely a modifiable risk factor for DPN and could modulate inflammatory mediators including IL-17 and IL-13 in DPN development [185]. Vitamin D supplementation relieved symptoms of neuropathy in T2D patients [186].

Nondiabetic pediatric OSA patients had reduced 25(OH)D levels [187], which may play a role in modulating the degree of insulin resistance and systemic inflammation [188]. Previous studies reported that OSA patients had lower vitamin D levels than healthy individuals [189–192]. Vitamin D can establish homeostasis between suppressor and regulatory T cell functions to modulate inflammatory process in OSA [193], suggesting that vitamin D deficiency in severe OSA patients is common, with a negative correlation between IL-17 and serum vitamin D levels [194]. Recent studies have shown that patients with OSA have a higher prevalence of vitamin D deficiency than healthy controls [190, 191, 195], and there are conflicting reports on the association between vitamin D deficiency and severity of OSA [191, 195–198]. PARP treatment may have late beneficial effects on vitamin D levels in selected OSA patients [199, 200]. Further studies exploring whether vitamin D deficiency may modulate OSA are needed.

#### 2.1.18. Neurotransmitters

Some neuromodulators such as glutamate, noradrenaline, acetylcholine, dopamine, and GABA could affect DPN and OSA. The effect on and association of the neurotransmitters with DPN and OSA are listed in Table 2.

#### 2.1.19. Glutamate

A previous study showed that the excitatory neurotransmitter glutamate induced an increased magnitude of mitochondrial depolarization, but no increase in apoptosis was observed [201]. In DPN, glutamate release is related with increased oxidative stress and decreased mitochondrial function, which is associated with neuropathic pain and activation of the glutamate recycling pathway that protects diabetic dorsal root ganglion by activating the SIRT1-PGC-1*α*-EFAM axis [202]. In OSA patients, higher glutamate levels were observed versus healthy subjects [203]. Similar changes on glutamate levels could lead to common mechanism in the pathogenesis of DPN and OSA.

#### 2.1.20. Noradrenaline

In OSA patients, nocturnal plasma noradrenaline content was increased and correlated with severity of overnight oxygen desaturation; 24 h urinary noradrenaline also increased [204]. In diabetic neuropathy rats, noradrenaline concentration was increased significantly 20 and 40 min after tramadol and clomipramine infusion [205], suggesting that the descending noradrenergic pathway could play an important role in analgesia for diabetic neuropathy. These findings indicate that noradrenaline could be involved in DPN and OSA.

#### 2.1.21. Acetylcholine

Diabetes was reported to impair acetylcholine-induced vascular relaxation in epineurial arterioles of the sciatic nerve [206]. A previous report showed that palmitic acid exposure could cause neuronal loss in diabetic neuropathy, which may be due to attenuated Ach synthesis [207]. In an OSA animal model, acetylcholine-induced vasodilation through the NO-dependent pathway in the skeletal muscle was impaired [208].

#### 2.1.22. Dopamine

A recent study showed that dopamine content was decreased in the midbrain, cerebral cortex, and brainstem regions, while it increased in the cerebellum and thalamus/hypothalamus in diabetic rats. No correlation between OSA and dopamine has been found [209].

#### 2.1.23. *γ*-Aminobutyric Acid (GABA)

In the adult nerve system, GABA is the main inhibitory neurotransmitter related with pain modulation. A previous study showed pronounced increase of extracellular GABA concentration in the ventromedial hypothalamic region in a type 1 diabetic animal model [210]. In diabetic neuropathy patients, GABA levels were significantly lower versus healthy controls [211]. The Toronto Expert Panel on Diabetic Neuropathy (TEPDN) recommended that GABA should be considered as first-line diabetic neuropathy treatment [212]. In OSA, low GABA levels were observed by“2-dimensional” spectroscopy [213] and reduced GABA levels were detected in the insular cortex of OSA patients [203]. The decrease in the levels of GABA in DPN and OSA has potential serious functional consequences that need to be elucidated.

## 3. Conclusions and Perspectives

**Figure 1:**
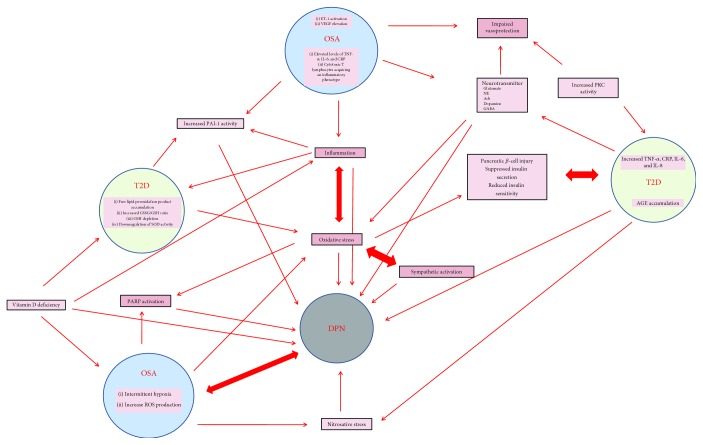
Pleiotropic interactions among type 2 diabetes (T2D), diabetic peripheral neuropathy (DPN), and obstructive sleep apnea (OSA).

Much of the data available on OSA and its association with DPN is difficult to interpret. The evidence reviewed in the current paper on the association between PDN and OSA in T2D patients suggests an intricate interrelationship among DPN, OSA, and T2D (Figure 1). OSA can aggravate and amplify T2D and subsequent complications such as DPN. Ultimately, OSA could contribute to T2D, resulting in a vicious circle. It is important to keep in mind that prospective studies with a larger T2D population are required to delineate the interwoven relationship between OSA and DPN. In addition, we summarized shared mechanisms in DPN and OSA, including oxidative stress, inflammation, AGEs, and PKC signaling. We also speculate that these mechanisms can operate simultaneously in patients with T2D leading to DPN and OSA. Recently, histone modifications are also reported to be involved in DPN and OSA. With the understanding of pathophysiology, CPAP is considered as the gold standard for managing patients with moderate to severe OSA. Further studies indicated that CPAP could improve insulin sensitivity in patients with prediabetes and decrease blood glucose in T2D patients; but till now, there has been no proof of efficacy of CPAP for DPN *in vivo* or *in vitro*. Similarly to CPAP, weight loss, bariatric intervention, or pharmacotherapy has been proven effective in alleviating OSA severity and improving glycemic status in obese T2D patients; future clinical trials will shed more light on the impact of CPAP therapy, weight loss, bariatric intervention, or pharmacotherapy on DPN. Prospective studies are required to determine mechanistic links applicable to DPN and OSA, which will contribute more to the exploration of novel therapeutic strategies in retarding the development and progression of DPN and OSA in T2D.

## Figures and Tables

**Table 1:** Summary of shared molecular mechanisms in DPN and OSA.

Molecular mechanisms	Subcategories	Reference for DPN	Reference for OSA
Oxidative stress		[76–80]	[71, 81–84, 93, 94]
Inflammatory markers	TNF-*α*	[101, 103]	[108–110, 113, 114]
	IL-6	[102]	[109–113]
	IL-8		[108]
	CRP	[103]	[111–113]
	NF-*κ*B	[104]	[85]
Endothelin-1 (ET-1)		[119]	[120–122, 126]
Vascular endothelial growth factor (VEGF)		[128]	[129, 130]
Advanced glycation end products (AGEs)		[138–142]	[146, 214, 215]
Protein kinase C (PKC)		[148]	[152]
Poly ADP ribose polymerase (PARP)		[153, 154]	[7, 8, 155–157]
Nitrosative stress	Nitrotyrosine	[162, 163]	[7]
Plasminogen activator inhibitor-1 (PAI-1)		[103]	[167–170]
Vitamin D deficiency		[176–179, 181–183, 185]	[187–192, 194–197]

DPN: diabetic peripheral neuropathy; OSA: obstructive sleep apnea.

**Table 2:** Summary of shared molecular mechanisms involved in DPN and OSA.

Common neuromodulators	Changes in DPN	Changes in OSA
Glutamate	Increased release [202]	High levels [203]
Noradrenaline	Increased levels [205]	Plasma and 24 h urinary noradrenaline increased [204]
Acetylcholine (Ach)	Attenuated Ach synthesis [206, 207]	Induce vasodilation [208]
Dopamine	Increased/decreased in different regions of the nerve system	Not correlated with OSA [209]
*γ*-Aminobutyric acid (GABA)	Decrease in diabetic neuropathy patients [211]	Reduced GABA levels in OSA [203]

DPN: diabetic peripheral neuropathy; OSA: obstructive sleep apnea.
